# Pheromonal and Behavioral Cues Trigger Male-to-Female Aggression in *Drosophila*

**DOI:** 10.1371/journal.pbio.1000541

**Published:** 2010-11-23

**Authors:** María de la Paz Fernández, Yick-Bun Chan, Joanne Y. Yew, Jean-Christophe Billeter, Klaus Dreisewerd, Joel D. Levine, Edward A. Kravitz

**Affiliations:** 1 Department of Neurobiology, Harvard Medical School, Boston, Massachusetts, United States of America; 2 Temasek Life Sciences Laboratories, 1 Research Link National University of Singapore, Singapore; 3 Institute of Medical Physics and Biophysics, Westfälische Wilhelms-Universität Münster, Münster, Germany; 4 Department of Biological Sciences, National University of Singapore, Singapore; 5 Department of Biology, University of Toronto at Mississauga, Mississauga, Ontario, Canada; University of Massachusetts, United States of America

## Abstract

Appropriate displays of aggression rely on the ability to recognize potential competitors. As in most species, *Drosophila* males fight with other males and do not attack females. In insects, sex recognition is strongly dependent on chemosensory communication, mediated by cuticular hydrocarbons acting as pheromones. While the roles of chemical and other sensory cues in stimulating male to female courtship have been well characterized in *Drosophila*, the signals that elicit aggression remain unclear. Here we show that when female pheromones or behavior are masculinized, males recognize females as competitors and switch from courtship to aggression. To masculinize female pheromones, a transgene carrying dsRNA for the sex determination factor *transformer* (*traIR*) was targeted to the pheromone producing cells, the oenocytes. Shortly after copulation males attacked these females, indicating that pheromonal cues can override other sensory cues. Surprisingly, masculinization of female behavior by targeting *traIR* to the nervous system in an otherwise normal female also was sufficient to trigger male aggression. Simultaneous masculinization of both pheromones and behavior induced a complete switch in the normal male response to a female. Control males now fought rather than copulated with these females. In a reciprocal experiment, feminization of the oenocytes and nervous system in males by expression of *transformer* (*traF*) elicited high levels of courtship and little or no aggression from control males. Finally, when confronted with flies devoid of pheromones, control males attacked male but not female opponents, suggesting that aggression is not a default behavior in the absence of pheromonal cues. Thus, our results show that masculinization of either pheromones or behavior in females is sufficient to trigger male-to-female aggression. Moreover, by manipulating both the pheromonal profile and the fighting patterns displayed by the opponent, male behavioral responses towards males and females can be completely reversed. Therefore, both pheromonal and behavioral cues are used by *Drosophila* males in recognizing a conspecific as a competitor.

## Abstract 2

By genetically manipulating both pheromonal profiles and behavioral patterns, we find that *Drosophila* males showed a complete reversal in their patterns of aggression towards other males and females

## Author Summary

As in other species, the fruit fly *Drosophila melanogaster* uses chemical signals in the form of pheromones to recognize the species and sex of another individual. Males typically fight with other males and do not attack females. While the roles of pheromonal and other sensory cues in stimulating courtship towards females have been extensively studied, the signals that elicit aggression towards other males remain unclear. In this work, we use genetic tools to show that masculinization of female pheromones is sufficient to trigger aggression from wild type males towards females. Surprisingly, males also attacked females that displayed male patterns of aggression, even if they show normal female pheromonal profiles, indicating that pheromones are not the only cues important for identifying another animal as an opponent. By simultaneously manipulating pheromones and behavioral patterns of opponents, we can completely switch the behavioral response of males towards females and males. These results demonstrate that not only pheromonal but also behavioral cues can serve as triggers of aggression, underlining the importance of behavioral feedback in the manifestation of social behaviors.

## Introduction

Aggression is a complex, innate behavior that likely evolved in the context of obtaining or defending resources [1]–[3]. Appropriate displays of aggression rely on the correct identification of potential competitors. In *Drosophila* as in most species, males fight with other males [4]–[7] and do not attack females. A wide variety of sexually dimorphic cues might be used by a male in directing agonistic rather than reproductive behavior towards another fly. As in other insect species, sex recognition in flies is strongly dependent on chemical communication, mediated by surface cuticular hydrocarbons that serve as pheromones [8]–[13]. *Drosophila* cuticular hydrocarbons (CH) are sexually dimorphic; female surfaces are characterized by dienes like (Z,Z)-7,11 heptacosadiene and (Z,Z)-7,11 nonacosadiene that act as aphrodisiacs [12],[14],[15], while male surfaces include (Z)-7 tricosene [11],[16],[17] and 11-cis-vaccenyl acetate (cVA) [18]–[20] act as anti-aphrodisiacs to other males. While the effects of CH (called “pheromones” in what follows) in courtship have been described in detail (reviewed in [8]), little is known about the roles of these substances in aggression. Pheromones that promote aggressive behavior have been identified in vertebrate and other invertebrate species [21]–[25], and cVA has been reported to modulate male aggressiveness in flies [26]. However, to what extent pheromonal or other cues are sufficient to trigger aggression in *Drosophila* remains largely unknown.

Although complex interactions between genes, environmental signals, and hormones ultimately influence the development and manifestation of social behaviors like aggression [27]–[30], the core circuitry involved appears to be pre-wired in the nervous system, as animals with no previous social experience can engage in normal agonistic encounters. Both males and females display aggression, but the specific behavioral patterns displayed are sexually dimorphic [31]–[33]: of greatest importance to the present work is that males “lunge,” in which they rise high on their hind legs and snap down hard on an opponent with their fore legs, while females display “head butt” and “shove” behaviors in which they do not rise above the horizontal. Rarer high-intensity patterns of behavior displayed by males include “boxing” and “tussling.” Finally, males establish hierarchical relationships, while females do not [33]. Recently, it has been shown that male and female patterns of aggression can be switched by manipulation of male and female splice variants of the *fruitless* (*fru*) gene [34]. Manipulations of *transformer* (*tra*), a splicing factor required for female development [35], also have been shown to switch male and female patterns of aggression [36]: inhibiting *tra* expression in the female nervous system leads to the display of male-like fighting patterns, while ectopic expression of *tra* in the male nervous system leads to the display of female-like fighting patterns in males. *tra*, in conjunction with a second gene, *tra-2*, mediates sexual differentiation by altering the splicing of *doublesex* and *fru*, which code for transcription factors responsible for regulating the morphological and behavioral aspects of sexual development [34],[35],[37]–[43].

In this work, we aimed to identify the cues used by males in identifying a conspecific as an opponent. Our strategy was to interfere with the expression of *transformer* by targeting a transgene carrying a dsRNA for *tra* (traIR) to different female tissues using the Gal4/UAS system. These masculinized females were paired with wild type Canton-S males in order to search for male aggressive responses. In parallel experiments, we asked whether it was possible to prevent aggression from a wild type male against another male by reciprocal manipulations in male flies. Our results show that by manipulating the pheromonal profiles and fighting patterns displayed by an opponent, male behavioral responses towards females and males can be completely reversed: wild type males fight rather than court when both pheromones and behavior are masculinized in females and court rather than fight when they are feminized in males. We propose that both pheromonal and behavioral cues can serve as key elements that allow *Drosophila* males to recognize a conspecific as a competitor.

## Results

**Figure 1:**
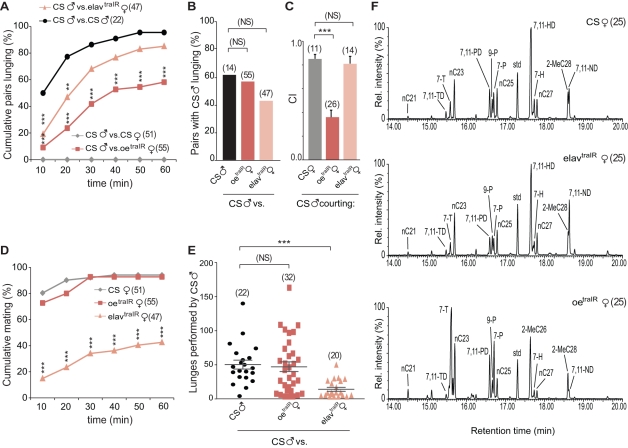
Masculinization of either pheromone profiles or fighting patterns in females triggers male aggression . (A–B) Fights between a CS male and an opponent scored for 1 h. Opponents are either a female of the indicated genotypes or another CS male. (A) Cumulative percentage of pairs that exhibit lunging (Chi-square test; ** *p*<0.01, *** *p*<0.001). (B) Percentage of pairs in which the CS male lunged at the opponent (Chi-square test; *p*>0.05). Pairs of control males were divided in two groups, according to paint color, and one was randomly chosen for scoring. (C) Male courtship towards decapitated female targets (ANOVA with Bonferroni post hoc test; *** *p*<0.001). (D) Percentage of CS males who mated with virgin females (Chi-square test; *** *p*<0.001). (E) Number of lunges performed by CS males. Each dot represents the number of lunges performed by one male (Mann Whitney test; *** *p*<0.001). (F) Cuticular hydrocarbons for each genotype were analyzed using gas chromatography mass spectrometry. The area of individual chromatographic peaks represents the abundance of a specific hydrocarbon species. Compared to controls and *elav* ^traIR^ females, *oe* ^traIR^ females exhibit significantly higher levels of male-characteristic alkenes (e.g., 7-T) and lower levels of female-associated pheromones 7,11-HD and 7,11-ND (Table 1). No significant differences were found between CS and *elav* ^traIR^ females. TD, tricosadiene; T, tricosene; PD, pentacosadiene; P, pentacosene; HD, heptacosadiene; H, heptacosene; ND, nonacosadiene. Error bars denote s.e.m.

Given the importance of pheromonal cues for sex recognition, we began by masculinizing the female oenocytes, specialized pheromone-producing cells [8],[11]. A transgene carrying a dsRNA for *tra* (*traIR*) was targeted to the oenocytes using an oenocyte-specific *Gal4* line [11]. These females were paired with wild type Canton-S in aggression assays. Surprisingly, pairings between wild type Canton-S males and *oeno-gal4/UAS-traIR* (*oe* ^traIR^) females revealed that masculinization of the pheromone profile elicits male aggression towards females (Figure 1A–B,E). For scoring, we quantified male lunging, as this is the most characteristic male aggressive response. Males never attacked wild type females (Figure 1A), even after copulation, when females display rejection behavior and have acquired some male CHs on their surfaces [44],[45]. In contrast, lunging behavior was observed in close to 60% of the experimental pairings, always performed by males (Figure 1B) since *oe* ^traIR^ females do not display lunging behavior (Figure S1A). The number of lunges directed towards *oe* ^traIR^ females was comparable to the number targeted at Canton-S males (Figure 1E). Male-to female aggression was never observed in fights between Canton-S males and any of the heterozygote parental control females either (*oeno-Gal4*/+ and *uas-traIR*/+ females; Figure S5B). Analysis by mass spectrometry (MS) of the CHs profile from both intact animals (Figure S2, see Methods) [45] and extract revealed that *oe* ^traIR^ females show a predominantly male profile, although small amounts of female CHs also are detected (Figure 1F, Table 1, and Figure S2). As expected, male-characteristic sex pheromones that are not produced by the oenocytes, namely cVA and the recently identified 3-O-acetyl-1,3-dihydroxy-octacosa-11,19-diene (CH503) [45], were not detected in females (Figure S2). These results demonstrate that partial masculinization of the female pheromonal profile is sufficient to trigger male-to-female aggression.

**Table 1:** GC-MS analysis of cuticular hydrocarbon extracts from control and masculinized females.

			Normalized Peak Area^2^: Females		
Retention Time (Min)	Compound^1^	Chemical Class	CS (*n* = 25)	elav^traIR^ (*n* = 25)	oeno^traIR^ (*n* = 25)
14.51	C21:0 (nC21)	alkane	0.66±0.06	0.49±0.10	2.79±0.28*
15.00	C22:1	monoene	0.08±0.02	0.05±0.01	1.13±0.40*
15.07	cVA	cVA	nd	nd	nd
15.11	C22:0	alkane	0.66 ±0.05	0.66±0.11	2.84±0.28*
15.48	7,11-C23:2 (7,11-TD)	diene	1.24±0.11*	0.9±0.13*	0.44±0.09*
15.54	9-C23:1	monoene	0.67±0.05	0.44±0.11	5.03±0.78*
15.59	7-C23:1 (7-T)	monoene	8.03±0.71	3.37±0.73	92.25±15.71*
15.65	5-C23:1	monoene	0.72±0.06	0.23±0.05	9.49±1.69*
15.69	C23:0 (nC23)	alkane	9.64±0.32	8.59±0.58	27.68±2.00*
16.05	C24:2	diene	0.16±0.03*	0.09±0.02*	0.058± 0.02*
16.13	C24:1	monoene	0.50±0.15	0.32±0.11	1.61± 0.74
16.25	C24:0	alkane	0.75±0.06*	0.82±0.04*	0.03±0.03 *
16.56	9,13-C25:2	diene	0.52±0.12*	0.40±0.07*	0.02±0.01*
16.60	7,11-C25:2 (7,11-PD)	diene	4.88±0.33**	3.02±0.46	2.34±0.47**
16.66	9-C25:1 (9-P)	monoene	6.32±0.53	3.71±0.63	6.94±1.46
16.70	7-C25:1 (7-P)	monoene	9.04±0.74	3.32±0.73	24.98±4.60*
16.75	5-C25:1	monoene	1.02±0.04	nd	1.26±0.22
16.79	C25:0 (nC25)	alkane	4.66±0.32	4.22±0.15	6.04±0.43*
17.12	C26:2	diene	0.60±0.07*	0.34±0.06*	0.21±0.06*
17.31	std (C26:0)	alkane	100	100	100
17.63	2-MeC26	Me-alkane	39.69±3.92	37.43±3.92	74.25±13.05*
17.66	7,11-C27:2 (7,11-HD)	diene	32.60±2.46	25.23±3.34	6.22±1.39*
17.70	9-C27:1	monoene	1.09±0.17	1.29±0.28	0.41±0.14
17.74	7-C27:1 (7-H)	monoene	4.48±0.52*	2.29±0.46	2.36±0.55
17.81	C27:0 (nC27)	alkane	2.15±0.25	2.48±0.18	2.20±0.30
18.29	C28:0	alkane	0.14±0.02	0.17±0.04	0.20±0.03
18.60	2-MeC28	Me-alkane	16.60±0.79	16.50±0.30	28.79±3.15*
18.63	7,11-C29:2 (7,11-ND)	diene	6.93±1.03	10.22±2.24	0.86±0.45*
18.77	C29:0	alkane	0.27±0.06	0.25±0.03	0.39±0.13
19.62	2-MeC30	Me-alkane	2.82±0.22	2.74±0.27	3.56±0.33

1 Elemental composition is listed as the carbon chain length followed by the number of double bonds. In some cases, the position of the double bonds could not be determined.

2 The signal intensity for each hydrocarbon species was determined by dividing the area of the peak for each of the measured hydrocarbons to the area of the peak for the standard. Even though the amount of standard is a known quantity, absolute quantitation is not possible with a single standard since compounds of different elemental compositions ionize differently. Hence, the ion signal reflects both (1) abundance and (2) volatility of the compound.

*p<0.05 when compared to the other two genotypes;

**p<0.05 when comparing CS versus *oeno* ^traIR^ (ANOVA followed by post hoc Tukey HSD test); nd, not detected.

Males consistently court decapitated wild type females, but they do not attack decapitated or immobilized males, suggesting that male pheromones can elicit aggression only in the context of a moving fly. This observation raised the question of whether behavior of another animal could also contribute to the triggering of aggression. We hypothesized that the display of male patterns of behavior by the opponent might stimulate aggressive responses from a male. To test this, we masculinized the female nervous system, by using the pan-neuronal driver *elav-Gal4*. This strategy has been shown to induce expression of FruM in the female CNS [36]. Moreover, it induced male-like patterns of fighting behavior in females; pairs of *elav-gal4;UAS-traIR* (*elav* ^traIR^) females are highly aggressive and lunge at each other [36]. We paired Canton-S males with behaviorally masculinized *elav* ^traIR^ females and found that 85% of these pairs showed lunging (Figure 1A). In this case, females lunged intensely at the males and initiated most of the fights (Figure S1). However, a smaller but substantial fraction of the males lunged at the females (Figure 1B), with a 3-fold reduction in the number of lunges compared to that performed towards *oe* ^traIR^ (Figure 1E). The fact that females usually dominate these fights (Figure S1B–C) is likely to be due to the fact that males persistently court the females despite being lunged at by them. The considerable difference in size between females and males also might contribute to giving the females an advantage [46],[47]. Male aggression towards females was not observed in fights between Canton-S males and any of the heterozygote parental control females (*oeno-Gal4*/+ and *uas-traIR*/+ females; Figure S5B). Since the pheromone profile of *elav* ^traIR^ is unaffected (Figure 1F, Table 1, and Figure S2), these females are as attractive as control females and males vigorously court them before transitioning to aggression. Nonetheless, because *elav* ^traIR^ females display aggressiveness towards the males, only 42% of these pairings resulted in successful copulation (Figure 1D). Courtship experiments towards headless targets confirm that in the absence of behavioral cues males cannot distinguish between *elav* ^traIR^ and Canton-S females (Figure 1C). Thus, males are willing to attack an opponent that exhibits male fighting behavior, even if that opponent is morphologically female and has a normal female pheromone profile.

**Figure 2:**
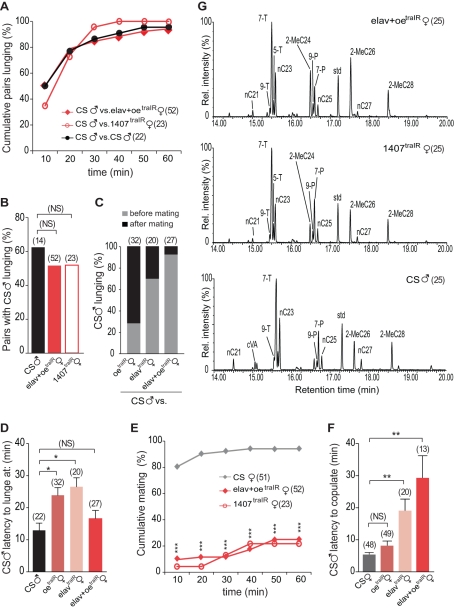
Simultaneous masculinization of pheromones and behavior invert normal male-female dynamics. (A) Cumulative percentage of pairs that exhibit lunging in fights between a CS male and either another CS male or a female of the indicated genotype. (B) Percentage of pairs in which CS males lunged at the opponent (Chi-square test; *p*>0.05). (C) Distribution of the male-to-female attacks. The bars represent the percentages of the males who lunged at each female before or after mating. In pairs that did not copulate but in which males lunged, the numbers are included in the group lunged before mating. (D) Latency to lunge at each opponent by control males. No significant differences were found in the CS male latency to lunge at *elav-Gal4;oeno-Gal4;UAS-traIR* (*elav*+*oe* ^traIR^) females compared to control males (ANOVA with Bonferroni post hoc test; * *p*<0.05). (E) Percentage of CS males that mated with virgin females of the indicated genotypes (Chi-square test; *** *p*<0.001). (F) Latency for the male to copulate with each type of female (ANOVA with Bonferroni post hoc test; ** *p*<0.01). Error bars denote s.e.m. (G) Cuticular hydrocarbons for each genotype were analyzed using gas chromatography mass spectrometry. The area of individual chromatographic peaks represents the abundance of a specific hydrocarbon species. TD, tricosadiene; T, tricosene; PD, pentacosadiene; P, pentacosene; HD, heptacosadiene; H, heptacosene; ND, nonacosadiene. Error bars denote s.e.m.

In order to analyze male responses towards further masculinized females, we simultaneously changed the sex of the female oenocytes and nervous system. When males were paired with *elav-gal4;oeno-gal4/UAS-traIR* females (*elav*+*oe* ^traIR^), lunging was observed in 94% of the fights (Figure 2A–B). Like *elav* ^traIR^ females, *elav*+*oe* ^traIR^ females initiated and dominated most fights (Figure S3A–C). Remarkably, 92% of the males who lunged at these females did so prior to or without ever copulating (Figure 2C). Since females do not make cVA, and this compound is only present on females after copulation, these results in which males attack females with masculinized hydrocarbon profiles but lacking cVA directly demonstrate that cVA is not necessary to trigger aggression. This is consistent with what was previously reported by Wang et al. [26], showing that cVA promotes aggression but it is not required to initiate it [26]. The male latency to lunge at *elav*+*oe* ^traIR^ females was similar to that of pairs of Canton-S males (Figure 2D). Moreover, successful copulation was observed in fewer than 25% of these pairings (Figure 2E) and the latency to achieve copulation was 6-fold higher compared to Canton-S females (Figure 2F). Thus, wild type males respond to *elav*+*oe* ^traIR^ females as potential competitors rather than as potential mates. As further confirmation of these observations, we expressed *traIR* under control of a *1407-gal4*, a line that drives expression both in the oenocytes [12],[48] and in the nervous system [48]–[52]. Expression of *uas-traIR* in females under the control of *1407-Gal4* has been previously shown by our laboratory to induce expression of FruM in the CNS [36], and pairs of *1407-gal4/UAS-traIR* (*1407* ^traIR^) females frequently lunge, although they show a mixture of male and female fighting patterns [36]. When paired with Canton-S males, *1407* ^traIR^ females were as aggressive as *elav*+*oe* ^traIR^ (Figure S3D–E), and the male response towards these two genotypes of females was indistinguishable (Figure 2A–B,E). All the observed pairs of Canton-S males with *1407* ^traIR^ females showed lunging (Figure 2A), and only 25% of them copulated throughout 1 h (Figure 2E). Analysis by MS of the CHs profile revealed that both *elav*+*oe* ^traIR^ and *1407* ^traIR^ females show a predominantly male profile (Figure 2G). Taken together, these results demonstrate that the display of both male pheromones and male patterns of behavior in a female reverses the normal dynamics between males and females.

**Figure 3:**
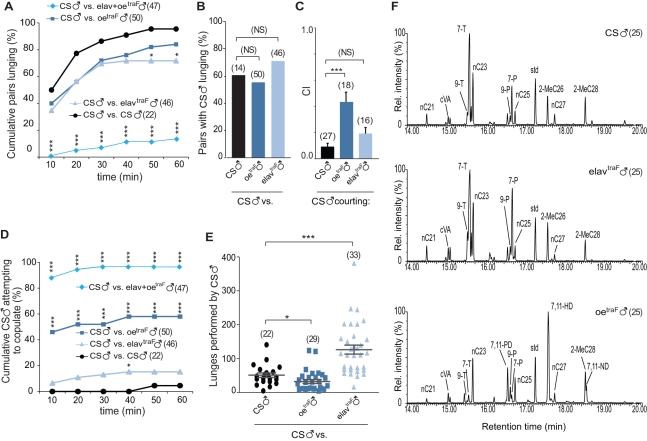
Feminization of pheromones and behavior in males inhibits aggression from wild type males. (A) Cumulative percentage of pairs that exhibit lunging. Fights are pairs between a CS male and a male of one of the indicated genotypes (Chi-square test; * *p*<0.05, *** *p*<0.001). (B) Percentage of pairs in which CS males lunged at the opponent. No significant differences were found compared to controls (Chi-square test; *p*>0.05). (C) CS male courtship towards headless male targets within 10 min (ANOVA with Bonferroni post hoc test; *** *p*<0.001). (D) Cumulative latency of CS males to attempt copulation (Chi-square test; * *p*<0.05, *** *p*<0.001). (E) Number of lunges performed by CS males. Each dot represents the total number of lunges performed by one CS male (Mann Whitney test; * *p*<0.05, *** *p*<0.001). (F) Cuticular hydrocarbons were analyzed using gas chromatography mass spectrometry. The area of individual chromatographic peaks represents the abundance of a specific hydrocarbon species. Compared to controls and *elav* ^traF^ males, *oe* ^traF^ males exhibit significantly higher levels of female-characteristic pheromones (e.g., 7,11-HD and 7,11-ND) and lower levels of alkanes and male-associated 7-T. Compared to CS males, *elav* ^traF^ males contained higher levels of alkanes and 7-T (Table 2). T, tricosene; PD, pentacosadiene; P, pentacosene; HD, heptacosadiene; ND, nonacosadiene. Error bars denote s.e.m.

We next asked whether it was possible to inhibit male aggression towards other males. We employed a symmetric strategy, feminizing the same tissues in males by expressing an active form of *transformer* (*traF*). Since males attack females that exhibit male pheromonal profiles but wild type female behavior (*oeno* ^traIR^; Figure 1B,E), suppression of male behavioral patterns by expressing *traF* in the nervous system should not prevent aggression from wild type males. Indeed, Canton-S males showed high intensity aggression towards *elav-gal4;UAS-traF* (*elav* ^traF^) males (Figure 3A–B,E). There was a substantial increase in the number of lunges that CS males directed to *elav* ^traF^ males compared to that directed towards both other Canton-S males (Figure 3E), despite the fact that *elav* ^traF^ males do not exhibit male patterns of aggression. Reciprocally, since the masculinization of the female nervous system triggers male aggression, the display of feminized pheromonal profiles in males should not completely suppress aggression from Canton-S males. Previous studies have shown that feminization of male pheromones elicits vigorous courtship behavior from wild type males [12]. Despite persistent courtship and frequent copulation attempts towards *oeno-gal4/UAS-traF* (*oe* ^traF^) males (Figure 3D), Canton-S males eventually transitioned to aggression. Canton-S males display normal aggression and courtship responses towards males from all the parental control lines (*elav-Gal4/*+, *oeno-Gal4/*+, and *uas-traF*/+ males; Figure S5). Courtship assays using headless target males confirm that *oe* ^traF^ males are highly attractive for CS males, since courtship index towards these males is significantly higher compared to courtship towards CS (Figure 3C). Mass spectrometric analyses revealed that *oe* ^traF^ males show reduced levels of (z)-7-tricosene and intense signals from diene hydrocarbons that are characteristic of females (Figure 3F, Table 2, and Figure S4). As expected, both control and experimental males still express cVA and CH503 (Figure S4).

**Table 2:** GC-MS analysis of cuticular hydrocarbon extracts from control and feminized males.

			Normalized Peak Area^2^: Males		
Retention Time (Min)	Compound^1^	Chemical Class	CS (*n* = 25)	elav^traF^ (*n* = 25)	oeno^traF^ (*n* = 25)
14.51	C21:0 (nC21)	alkane	2.81±0.11*	3.98±0.23*	1.75±0.18*
15.00	C22:1	monoene	1.21±0.12	2.36±0.27*	0.27±0.07
15.07	cVA	cVA	4.1±0.12	4.38±0.92	4.29±1.12
15.11	C22:0	alkane	1.72±0.22	3.46±0.28*	1.66±0.12
15.48	7,11-C23:2	diene	0.01±0.003	0.01±0.007	2.08±0.20*
15.54	9-C23:1 (9-T)	monoene	4.98±0.87	15.91±0.77*	2.33±0.63
15.59	7-C23:1 (7-T)	monoene	89.32±4.20*	147.55±13.82*	16.91±5.61*
15.65	5-C23:1	monoene	7.68±0.43	19.73±2.83*	1.81±0.55
15.69	C23:0 (nC23)	alkane	18.27±0.56	32.89±2.27*	17.68±1.56
16.05	C24:2	diene	nd	nd	0.31±0.07*
16.13	C24:1	monoene	2.1±0.38	6.49±0.70*	0.77±0.48
16.25	C24:0	alkane	0.43±0.05*	0.89±0.10*	1.13±0.13*
16.56	9,13-C25:2	diene	nd	nd	0.60±0.11*
16.60	7,11-C25:2 (7,11-PD)	diene	nd	nd	7.74±0.63*
16.60	2-MeC24	Me-alkane	7.54±0.60	19.68±3.34*	10.66±2.29
16.66	9-C25:1 (9-P)	monoene	2.94±0.44***	8.35±1.21***	7.74±1.77
16.70	7-C25:1 (7-P)	monoene	13.71±1.29	70.78±10.32*	12.04±4.41
16.75	5-C25:1	monoene	0.30±0.03***	2.37±0.79***	1.19±0.25
16.79	C25:0 (nC25)	alkane	2.24±0.11*	3.39±0.43*	4.91±0.45*
17.12	C26:2	diene	nd	nd	0.66±0.06*
17.31	std (C26:0)	alkane	100	100	100
17.63	2-MeC26	Me-alkane	23.68±2.31***	58.97±8.92***	53.25±10.33
17.66	7,11-C27:2 (7,11-HD)	diene	nd	nd	29.03±1.28*
17.70	9-C27:1	monoene	0.03±0.01	0.10±0.05	0.97±0.26
17.74	7-C27:1	monoene	0.27±0.05**	1.17±0.34	2.72±0.92**
17.81	C27:0 (nC27)	alkane	1.35±0.11	1.13±0.17^†^	2.30±0.39^†^
18.29	C28:0	alkane	0.11±0.03	0.13±0.04	0.20±0.07
18.60	2-MeC28	Me-alkane	19.97±1.77	23.55±3.07	24.83±2.77
18.63	7,11-C29:2 (7,11-ND)	diene	nd	nd	5.93±0.96*
18.77	C29:0	alkane	0.34±0.06	0.17±0.02	0.29±0.08
19.62	2-MeC30	Me-alkane	3.01±0.37	1.46±0.14	1.94±0.59

1 Elemental composition is listed as the carbon chain length followed by the number of double bonds. In some cases, the position of the double bonds could not be determined.

2 The signal intensity for each hydrocarbon species was determined by dividing the area of the peak for each of the measured hydrocarbons to the area of the peak for the standard. Even though the amount of standard is a known quantity, absolute quantitation is not possible with a single standard since compounds of different elemental compositions ionize differently. Hence, the ion signal reflects both (1) abundance and (2) volatility of the compound;

*p<0.05 when compared to the other two genotypes;

**p<0.05 when comparing CS versus *oeno* ^traF^;

***p<0.05 when comparing CS versus *elav^t^* ^raF^;

****p<0.05 when comparing *oeno* ^traF^ versus *elav^t^* ^raF^ (ANOVA followed by post hoc Tukey HSD test); nd, not detected.

We next asked whether simultaneous feminization of oenocytes and the nervous system in males was sufficient to prevent aggression from wild type males. Indeed, males expressing *traF* driven by both *elav-gal4* and *oeno-gal4* trigger responses in males that are opposite to those anticipated in normal male-male interactions. Analysis by MS of the CH profile revealed that *elav*+*oe* ^traF^ males show a predominantly female profile (Figure S6). Aggression towards these males was greatly reduced, since in only 6 out of the 47 pairs analyzed did Canton-S males attack them (Figure 3A). The fact that some *elav*+*oe* ^traF^ males were still attacked is likely due to the presence of residual male pheromones (Figure S6). Remarkably, 96% of the Canton-S males persistently courted and attempted copulation with *elav*+*oe* ^traF^ males (Figure 3D). These effects were significantly different from those obtained with *oe* ^traF^ males and resembled the normal responses of males towards females.

**Figure 4:**
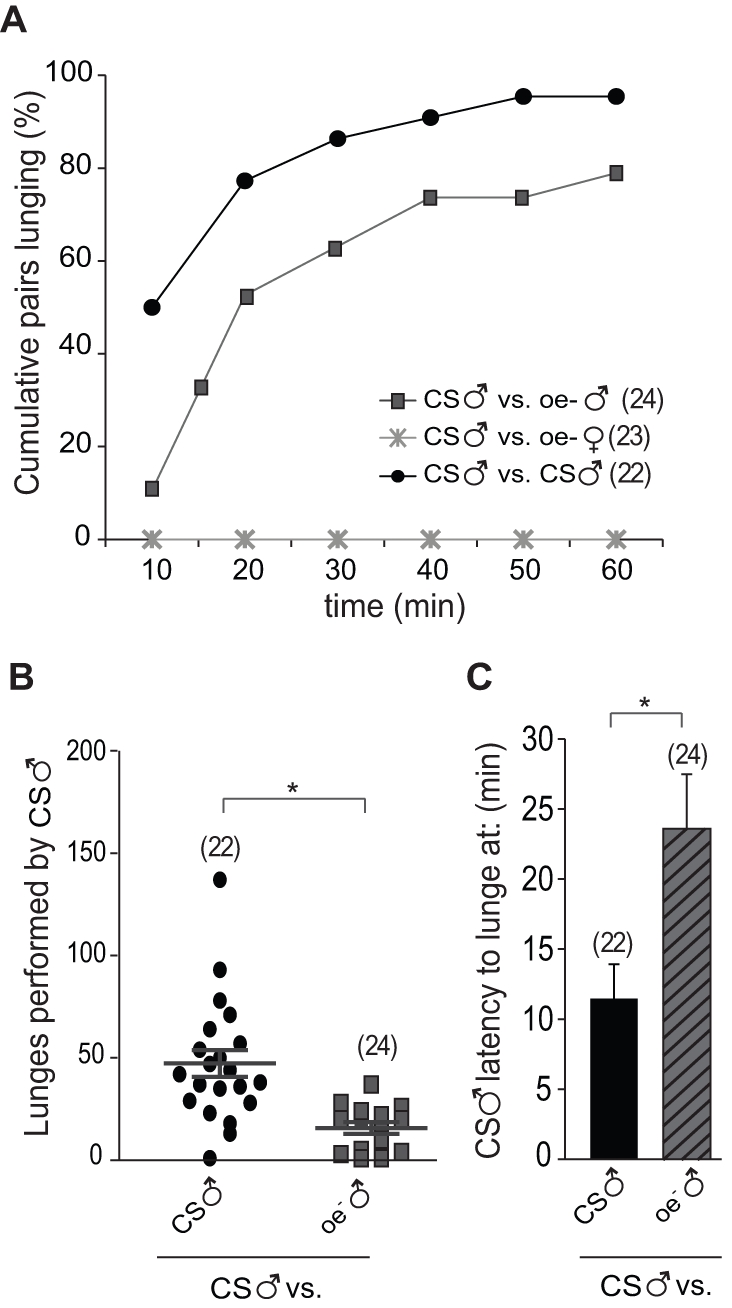
Control males do not attack oenocyte-less females. (A) Cumulative percentage of pairs that exhibit lunging in fights between CS males and an oenocytes-less (*oe* ^−^) male or female opponent. Aggression towards *oe* ^−^ females was never observed. (B) Number of lunges performed by CS males towards another CS male or a *oe* ^−^ male. Each dot represents the total number of lunges performed by one CS male during a fight (Mann-Whitney test; * *p*<0.05). (C) Latency of CS males to lunge at each category of opponent (Student's *t* test; * *p*<0.05). Error bars denote s.e.m.

Previous experiments using oenocyte-less (*oe* ^−^) flies showed that males court both males and females that are devoid of CHs [11], suggesting that courtship is a “default” behavior in the absence of pheromonal cues. If aggression is also a default behavior, which is normally suppressed by female pheromones, wild type males should attack both *oe* ^−^ male and *oe* ^−^ female opponents. If instead aggression has to be triggered actively either via pheromonal or behavioral cues, males should not attack *oe* ^−^ flies that do not display male behavior. Indeed, aggression assays showed that Canton-S males did not display aggressive behavior towards *oe* ^−^ females (Figure 4). In contrast, they did attack *oe* ^−^ males (Figure 4A,B), although at a reduced intensity compared to controls (Figure 4B,C). Reduced aggressiveness directed towards *oe* ^−^ males indicates that pheromones missing from these males are required for normal intensity levels of fighting. It should be noted that *oe* ^−^ males still have normal levels of cVA [11], which could also contribute to the aggressiveness displayed towards them by Canton-S males. Like *oe* ^traIR^ females, *oe* ^−^ females show wild type behavior and copulate with males. Nevertheless, males did not attack *oe* ^−^ females, even when they had previously mated with other males (unpublished data). Future experiments will attempt to identify the male pheromonal cues that are sufficient to trigger male aggression against opponents who show no aggression towards them.

Results presented here demonstrate that intense male aggression is evoked when females display masculinized pheromonal profiles. They show further that cVA is not required to trigger aggression. Our results indicate that surface pheromonal cues eventually triumph over other sensory cues, since males ordinarily do not fight females. Surprisingly, males also attack any opponent, male or female, displaying male behavior. The fact that males do not attack *oe* ^−^ females but do attack *oe* ^traIR^ and *elav* ^traIR^ females suggests that, unlike courtship, aggression is not a default behavior and has to be actively triggered. The stimuli may be either chemical cues, which would be perceived through chemosensory input pathways, or cues derived from the display of male behavioral patterns, probably perceived via multimodal input pathways. The male willingness to attack *elav* ^traIR^ females, which exhibit normal female pheromone profiles, is an unexpected result that could be accounted for by different scenarios. Males could be responding to a specific cue that triggers lunging behavior as a stimulus-response effect (like a visual threat). However, this seems unlikely since we did not observe any specific behavioral pattern in females preceding attacks from Canton-S males. Alternatively, multiple cues emerging from the behavior of these aggressive females could be perceived by the males, converging on central neural pathways that ultimately determine the male switch from sexual to aggressive responses. Our results support the notion that whereas courtship is a default behavior, the escalation to aggressive interactions is a complex behavioral response that requires integration of different sensory modalities by higher order processing centers in the male brain.

In this work, we show that masculinization of either pheromones or behavior in females is sufficient to trigger male-to-female aggression. In support of this, feminization of only one of these factors in males is not sufficient to prevent aggression from Canton-S males. However, males display little or no aggression against males in which the pheromone profiles and fighting patterns were simultaneously feminized. Remarkably, genetically inverting male and female fighting patterns and pheromone profiles of an opponent is sufficient to completely *switch* the behavioral response of a male. Taken together, our results indicate that *Drosophila* males use pheromonal and behavioral cues to recognize a conspecific as a potential competitor.

## Materials and Methods

### Fly Rearing

All fly strains were reared on standard fly food (medium containing agar, glucose, sucrose, yeast, cornmeal, wheat germ, soya flour, molasses, propionic acid, and Tegosept). Flies were grown in temperature- and humidity-controlled incubators (25 °C, 50% humidity) on a 12-h light/dark cycle, except for the oenocyte-less flies. Male or female pupae were isolated approximately 24 h prior to eclosion and housed in individual vials with food medium for 6 d prior to use in experiments. In male-male fights, a small dot of a water-based acrylic paint was applied to the dorsal thorax so that individuals could be easily identified. This procedure was performed under CO2, at least 1 d before fighting.

### Strains and Crosses

Wild-type Canton-S and *elav^C155^-Gal4* lines were obtained from the Bloomington Stock Center. *uas-traIR* line was obtained from Barry Dickson (Vienna Drosophila RNAi Center, No.2560) and *uas-TraF* line was obtained from Bloomington Stock Center (No. 4590). The line *oeno-Gal4 (PromE(800,) line 2M)* was generated by J-C.B. [11]. We crossed either *elav^C155^-Gal4* or *oeno-Gal4* virgin females to males from the respective *uas* lines to generate the feminized or masculinized experimental lines. All the transgenes employed in each case were tested in heterozygosis (hemizygosis for males containing *elav-Gal4*). Adults lacking oenocytes were obtained as previously described [11]. For behavioral assays, all target flies generated in these cases had w^+^ background. We also used *1407-Gal4* (Bloomington No. 8751) to generate masculinized females as described in previous studies [36].

### Gas Chromatography MS Analysis

For each genotype, five flies were placed in 100 ml of hexane containing 10 mg/ml of synthetic hydrocarbon (hexacosane; Sigma-Aldrich) for 30 min at room temperature. Five replicate samples were prepared for each genotype. The extract was removed, placed in a clean glass vial, and the solvent evaporated under vacuum. The extracts were re-dissolved in 30 ml of heptane prior to GC-MS analysis. GC-MS analysis was performed with a Quattromicro-GC (Waters, Manchester, UK) equipped with a HP-5 (5%-Phenyl-methylpolysiloxane column; 30 m length, 0.32 mm ID, 0.25 µm film thickness; Agilent). Ionization was achieved by electron ionization (EI) at 70 eV. One ml of the sample was injected using a splitless injector. The helium flow was set at 1.3 ml/min. The column temperature program started at 50 °C for 2 min, then increased to 300 °C at a rate of 15 °C/min. The quadrupole mass spectrometer was set to unit mass resolution and 3 scans/min, from m/z 37 to 700. Chromatograms and mass spectra from GC-MS analysis were analyzed using MassLynx (Waters, Manchester, UK). Compounds were identified on the basis of retention time and EI mass spectra. To determine the signal intensity for each hydrocarbon species, the area of its chromatographic peak from the total ion chromatogram was calculated and normalized to the area of the signal corresponding to the synthetic standard. Statistical analysis was performed using analysis of variance (ANOVA) followed by post hoc analysis with a Tukey-Kramer honestly significant difference (HSD) test (http://faculty.vassar.edu/lowry/VassarStats.html).

### Behavioral Assays

Aggression and courtship (male-female or male-male) assays were performed in individual chambers of 12-well polystyrene plates (each chamber dimension is 10 mm diameter × 5 mm depth) containing a food cup made of the cap of a 1.5 ml Eppendorf tube. Flies were transferred in pairs to assay chambers by aspiration. Experiments were started at *Zeitgeber* time 1 at 25 °C in a humidity controlled room (50%). For quantification of courtship towards decapitated targets, headless flies were placed in the center of the food cup prior to the transfer of the courting CS males. The courtship index is the fraction of a 10-min observation period spent by the male exhibiting courtship steps such as tapping, wing extension, licking, and attempting copulation, starting from the onset of courtship. The same chambers and conditions were used for courtship and aggression experiments to allow comparisons between experiments, since differences in chamber size lead to variations in behavior. Fights and courtship assays were videotaped and tapes were scored blindly. Courtship assays were recorded for 20 min while aggression assays were videotaped for 90 min and scored for 60 min after the time when both flies were introduced to the chamber.

Latency to court, attempted copulation, and mating with intact targets were determined from recordings of the aggression assays. The time between when flies were loaded and the onset of copulation was defined as the mating latency. Similar criteria were used for determining courtship latency and attempted copulation latency. Attempted copulation is scored when courting males bend their abdomens towards the courtship object. For aggression assays, pairs of a *Canton-S* male and either a male or a female opponent were placed in each chamber. Lunging behavior was determined as previously described [6]. The time between when flies were loaded into chambers and the first lunge displayed by CS males was defined as the latency to lunge.

### Statistical Analysis

Statistical analyses were performed with the Prism software (version 5.0b, SPSS Inc.). *p* values were determined either via two-tailed Student's *t* test when comparing two groups or via ANOVA followed by the post hoc Bonferroni test when comparing multiple groups. For data that did not follow a parametric distribution, Mann-Whitney test was used for comparing two groups.

## Supporting Information

Figure S1 **Behaviorally masculinized females are highly aggressive in dyadic encounters with males.** (A) Percentage of females of each of the indicated genotypes who lunged when paired with a control male. (B) Average percentage of lunges performed by each opponent in fights between control males and *elav* ^traIR^ females (Student's *t* test; *** *p*<0.001). (C) Percentage of the fights between control males and *elav* ^traIR^ females that was initiated by each opponent, where initiation is defined as being the first one to lunge. Asterisks indicate significant differences between genotypes as determined by a chi-square test (*** *p*<0.001). Error bars denote s.e.m.
(0.42 MB EPS)

Figure S2 **Representative UV-LDI mass spectra recorded from the anogenital (AG) region of control and masculinized females.** (A) Profile of CS female AG region. (B) The cuticular profile of *elav* ^traIR^ females is qualitatively similar to the profile from CS females. (C) The cuticules of *oeno* ^traIR^ females exhibit a mixture of diene hydrocarbons (characteristic of females) and high levels of characteristic male hydrocarbons (highlighted in blue). All assigned signals correspond to potassiated molecules [M+K]+. Peaks corresponding to sodiated molecules of the same hydrocarbon species are not labeled.
(0.52 MB EPS)

Figure S3 **Behaviorally and pheromonally masculinized females dominate fights with wild type males.** (A–C) Fights between control males and *elav+oe* ^traIR^ females. (A) Average percentage of lunges performed by each opponent (Student's *t* test, *** *p*<0.001). Error bars denote s.e.m. (B) Percentage of fights that were initiated by each opponent. A significant difference was determined by a chi-square test (*** *p*<0.001). (C) Percentage of fights in which either one or both opponents showed lunging behavior. (D–E) Fights between control males and *1407* ^traIR^ females. (D) Percentage of the fights initiated by each opponent (Chi-square test; *** *p*<0.001). (E) Percentage of fights where only one opponent, or both, showed lunging behavior.
(0.50 MB EPS)

Figure S4 **Representative UV-LDI mass spectra recorded from the legs and anogenital region (AG) of Canton S and experimental males.** (A–B) Spectra from Canton-S males. (C–D) Spectra from males in which *traF* is ectopically expressed using the pan-neural driver *elav-Gal4*. (E–F) Spectra from males in which *traF* is ectopically expressed using the oenocyte-specific driver *oeno-Gal4*. These males contain a mixture of characteristic male CHs (e.g., oxygen-containing alkenes) in addition to high levels of characteristic female CHs (highlighted in red). The male sex-pheromones cVA and CH503 are present in the AG region of all three genotypes. Compounds other than CHs such as fatty acids and oligosaccharides are also detected. All assigned signals correspond to potassiated molecules [M+K]+. Peaks corresponding to sodiated species are not labeled.
(0.81 MB EPS)

Figure S5 **Canton-S males show normal behavioral responses towards both female and male parental control lines.** (A–C) Fights between CS males and either a male (*elav-Gal4* hemizygote, *oeno-Gal4* heterozygote, or *uas-traF* heterozygote) or a female (*elav-Gal4* heterozygote, *oeno-Gal4* heterozygote, or *uas-traIR* heterozygote) of the indicated parental control lines. (A) Cumulative number of pairs where any of the opponents showed lunging. In pairings between Canton-S males and each of the parental control females, lunges were never observed by any of the opponents. No significant differences were found in fights between a Canton-S male and any of the parental control lines compared to fights between two Canton-S males (Fisher's exact test; *p*>0.05). (B) Percentage of fights where CS males lunged at the opponent. In none of the pairings analyzed did Canton-S males lunge at any of the females. Canton-S males never attacked heterozygote *1407-Gal4*/+ females either (unpublished data). No significant differences were found in the percentage of Canton-S males that lunged at any of the analyzed lines (either experimental or control lines, including CS and all of the heterozygote parental control lines) (Fisher's exact test; *p*>0.05). (C) Number of lunges directed by Canton-S males towards males of the indicated genotypes. No significant differences were found between the number of lunges towards another CS male and the number of lunges directed towards any of the heterozygote parental control males (Mann-Whitney test; *p*>005). (D) Cumulative percentage of CS males that mated with virgin females of the indicated genotypes. (E) Percentage of CS males that attempted to copulate with males of the indicated genotypes throughout the 1-h fight. (F) Male courtship towards decapitated female targets. No significant differences were found in courtship index towards any of the control females, including *1407-Gal4*/+ females (ANOVA with Bonferroni post hoc test; *p*>0.05). No significant differences were found in CI towards *elav* ^traIR^ females and CI towards its heterozygote parental control lines (*elav-Gal4*/+ and *uas-traIR*/+ females; ANOVA with Bonferroni post hoc test; *p*>0.05). CI towards *oeno* ^traIR^ females was significantly lower than CI towards its heterozygote parental control lines (*oeno-Gal4*/+ and *uas-traIR*/+ females; ANOVA with Bonferroni post hoc test; *p*<0.001 and *p*<0.001, respectively). (G) Male courtship towards decapitated male targets. No significant differences were found in courtship index towards any of the male targets (*p*>0.05, ANOVA with Bonferroni post hoc test). No significant differences were found in CI towards *elav* ^traF^ males and CI towards its heterozygote parental control lines (*elav-Gal4*/+ and *uas-traIR*/+ males; ANOVA with Bonferroni post hoc test; *p*>0.05). CI towards *oeno* ^traF^ males was significantly higher than CI towards its heterozygote parental control lines (*oeno-Gal4*/+ and *uas-traIR*/+ males; ANOVA with Bonferroni post hoc test; *p*<0.01 and *p*<0.001, respectively). Error bars denote S.E.M.
(0.59 MB EPS)

Figure S6 **Cuticular hydrocarbons for each genotype were analyzed using gas chromatography mass spectrometry.** The area of individual chromatographic peaks represents the abundance of a specific hydrocarbon species. Compared to controls, both *1407* ^traF^ and *elav+oeno* ^traF^ males exhibit significantly lower levels of male-characteristic alkenes (e.g., 7-T) and higher levels of female-associated pheromones 7,11-HD and 7,11-ND. TD, tricosadiene; T, tricosene; PD, pentacosadiene; P, pentacosene; HD, heptacosadiene; H, heptacosene; ND, nonacosadiene.
(0.60 MB EPS)

## Abbreviations

CH: cuticular hydrocarbon
*fru*: *fruitless*
*tra*: *transformer*
MS: mass spectrometry
*traF*: *transformer*
