# Correction: Pheromonal and Behavioral Cues Trigger Male-to-Female Aggression in *Drosophila*

**DOI:** 10.1371/annotation/1c19d040-9f9f-4b9f-b678-70f1fe387192

**Published:** 2010-12-02

**Authors:** María de la Paz Fernández, Yick-Bun Chan, Joanne Y. Yew, Jean-Christophe Billeter, Klaus Dreisewerd, Joel D. Levine, Edward A. Kravitz

The first author's name is cited incorrectly. The correct citation is: Fernández MP, Chan Y-B, Yew JY, Billeter J-C, Dreisewerd K, et al. (2010) Pheromonal and Behavioral Cues Trigger Male-to-Female Aggression in Drosophila. PLoS Biol 8(11): e1000541. doi:10.1371/journal.pbio.1000541

